# Recognition of Loss & Damage from wildfires is key for climate justice

**DOI:** 10.1038/s43247-025-02968-w

**Published:** 2025-11-14

**Authors:** Renata M. da Veiga, Maria L. F. Barbosa, Fiona R. Spuler, Igor J. M. Ferreira, Julia Mindlin, Douglas I. Kelley, Victoria Matusevich, Regina R. Rodrigues, Ane Alencar, Daniel C. Ratilla, Liana O. Anderson, Michel Valette, Renata Libonati, Rodrigo A. Estevez, Tainan Kumaruara, Caroline Camilo Dantas, Santiago I. Hurtado

**Affiliations:** 1 Department of Meteorology, Federal University of Rio de Janeiro, Rio de Janeiro, Brazil; 2 Water and Climate Science, UK Centre for Ecology and Hydrology, Wallingford, Oxfordshire UK; 3 Department of Meteorology, University of Reading, Reading, UK; 4 TRopical Ecosystems and Environmental Sciences lab, Earth Observation and Geoinformatics Division, National Institute for Space Research, São José dos Campos, Brazil; 5 Leipzig Institute for Meteorology, Leipzig University, Leipzig, Germany; 6 Fundación Avina, Ciudad del Saber, Clayton, Ciudad de Panamá, Panamá; 7 Department of Oceanography, Federal University of Santa Catarina, Florianópolis, Brazil; 8 Amazon Environmental Research Institute (IPAM), Brasília, Brazil; 9 Ateneo Institute of Sustainability, Ateneo de Manila University, Quezon City, Philippines; 10 Department of Environmental Science, Ateneo de Manila University, Quezon City, Philippines; 11 Leverhulme Centre for Wildfires, Environment, and Society, London, UK; 12 Centre for Environmental Policy, Imperial College London, London, UK; 13 Centro de Investigación e Innovación para el Cambio Climático, Facultad de Ciencias, Universidad Santo Tomás, Santiago, Chile; 14 Instituto Milenio en Socio-Ecología Costera, Santiago, Chile; 15 Brigada Guardiões Kumaruara, Baixo Tapajós - Resex Tapajos Arapiuns, Pará, Brazil; 16 Instituto Cafuringa, Rua 4 travessa 4 entrada 2 ch: Rocha sagrada encantaria, Brasilia-DF, Brazil; 17 Brigada Voluntária Guardiões da Cafuringa, APA da Cafuringa/Núcleo Rural Lago Oeste, Brasília-DF, Brazil; 18 Consejo Nacional de Investigaciones Científicas y Técnicas (CONICET), Centro Científico Tecnológico Patagonia Norte, Bariloche, Argentina

**Keywords:** Climate-change adaptation, Governance, Climate-change policy, Interdisciplinary studies

## Abstract

Wildfires are becoming one of the defining climate-related crises of the twenty-first century. We argue that their inclusion in the Loss & Damage framework of the United Nations Framework Convention on Climate Change is essential to support prevention, recovery and justice for the most affected communities.

Across continents, fire seasons are lengthening, extreme events are intensifying, and their cascading impacts on ecosystems, health, economy and livelihoods are expanding. Rising temperatures, prolonged droughts, and more frequent heatwaves boosted by anthropogenic climate change, together with growing land cover changes and poor land management, are increasing wildfire risk, occurrence, intensity and frequency globally^1–3^. Yet, despite the growing threat and scientific evidence that climate change is amplifying fire risk, wildfire impacts remain largely absent from international climate policy debates, particularly within the Loss & Damage agenda established under the United Nations Framework Convention on Climate Change (UNFCCC). This omission is increasingly damaging.

The escalating impacts of increasing wildfire activity, exacerbated by anthropogenic climate change, are driving profound economic and non-economic losses that disproportionately affect vulnerable populations. As such, including wildfires as a core component of the Loss & Damage framework is not only a scientific imperative but also a matter of climate justice.

The first call for proposals under the Fund for Responding to Loss & Damage is expected to be launched at the 30th Conference of the Parties in Belém, Brazil (COP30) in November 2025. We urge the explicit recognition of the drivers and impacts of wildfires in the Loss & Damage framework, along with targeted guidance under the Santiago Network^4^ to help with implementation. Only with full recognition can we ensure support for wildfire prevention, response, and long-term ecological and cultural resilience.

## Perspectives from Brazil

Representatives of local volunteer and community fire brigades, members of Indigenous Peoples and Local Communities, wildfire and climate scientists, policymakers, and climate finance experts convened in Brasilia in September 2025 for a multi-stakeholder workshop to identify critical gaps in wildfire governance, funding mechanisms, and justice-oriented approaches to climate risk.

Evidence from Brazil demonstrates the urgency of the task. In the northeastern Amazon, human-induced climate change has increased the likelihood of extreme fire weather by 30–70 times, leading to approximately four times more burned area than would have occurred without climate change^5^. In the Pantanal, such fire-prone conditions became 4–5 times more likely, resulting in approximately a 35-fold increase in burned area^5^.

Reports from Indigenous Peoples and Local Communities in meetings before and during the workshop in Brasilia highlighted that the timing and intensity of the fire season have shifted, the dry season has lengthened, health conditions have worsened and food insecurity has increased, which confirms science findings on changes in dry season patterns^5,6^. The loss of lands belonging to Indigenous Peoples and Local Communities, along with the decline of their traditional patch-based fire practices, which historically helped limit fire spread and preserve landscape connectivity, also plays a role in exacerbating the climate crisis^7^.

These stressors are linked to intensified wildfire regimes that disrupt Indigenous Peoples and Local Communities’ livelihoods, including fire practices. They thereby threaten the inheritance of traditional ecological knowledge^3^, because cumulative understandings, beliefs and practices are developed by local communities through long-term interactions with their environments. This knowledge system encompasses, for example, detailed insights into local ecosystems, seasonal patterns, and use of natural resources, all of which are transmitted across generations through observation, experience, and cultural traditions. One example that Indigenous Peoples and Local Communities noted is that natural patterns and indicators used to anticipate rainfall are no longer reliable, making it increasingly difficult to determine the best timing for the traditional use of fire.

Efforts and advances are being made in the Brazilian context. Although federal legislation has a history of disregarding the ecological and cultural aspects of fire^8^, Brazil has recently approved the Integrated Fire Management National Policy (Law 14,944/2024), which aims to promote fire management through an interdisciplinary approach, reducing the damages of wildfires nationally, recognizing the ecological role of fire, and respecting traditional knowledge and practices.

Already for centuries before the approval of the National Policy, Indigenous Peoples and Local Communities across Brazil have self-organized. In response to increased wildfire activity, they have established local volunteer and community brigades, provided environmental education among their communities, and have been managing their use of fire to account for the context of climate change. However, these efforts are chronically underfunded and lack institutional support, such as recognition of community firefighting as a formal profession and access to equipment, training, and adaptive resources.

Participants in the workshop highlighted the persistent gap in funding for wildfire prevention and preparedness. Fire suppression receives intermittent support during critical wildfire periods, but prevention measures, including early warning systems and community-based monitoring and management, are often neglected. Prioritizing prevention is key to minimizing the occurrence of intense wildfires^9^. By contrast, suppression of ongoing fires is far more demanding in terms of both human and financial resources. The structural barriers to accessing climate finance, especially through the UNFCCC’s Loss & Damage process, are even more pronounced than for mitigation and other adaptation projects. As a result, frontline communities are left to face the impacts without meaningful assistance.

## Wildfires drive losses and damages globally

Fire impacts extend beyond Brazil. Fire seasons in Australia, Africa, Southern Europe, Canada, and the United States over the past years have cost billions of dollars in damages, thousands of deaths from smoke exposure, and large-scale biodiversity loss^4^. These events show that wildfires are a global driver of climate-induced losses and damages that extend beyond economic metrics: they threaten ecosystems, public health, and cultural heritage worldwide. Wildfires demand urgent international attention.

Nevertheless, wildfire remains largely invisible within the current Loss & Damage framework, and it will likely be up to countries themselves to decide what funding requests under the framework will focus on. The establishment of the Loss & Damage Fund at the 28^th^ Conference of the Parties in Dubai (COP28) and the operationalization through the Santiago Network offer a pathway to redress. However, without explicit recognition of wildfires and their impacts, these mechanisms are incomplete, and we risk a perpetuation of blind spots in resource allocation and technical assistance.

Dedicated mechanisms for wildfires must be incorporated within the Loss & Damage process. This is particularly important because wildfires do not affect ecosystems and communities equally. Indigenous Peoples and Local Communities, whose livelihoods depend heavily on natural resources and fire use, are severely impacted by extensive wildfires and uncontrolled agricultural fires. They experience a disproportionate share of climate-related losses and damages. These include economic and non-economic losses: destroyed property limits access to infrastructure; damaged agricultural fields hinder food security; smoke affects physical health; and social isolation and erosion of traditional knowledge result in mental and emotional health issues.

## Wildfire support is a matter of climate justice

Vulnerable groups, such as Indigenous Peoples and Local Communities, have contributed the least to climate change but encounter systemic barriers that hinder their participation in political decision-making and access to funding. Both the distribution of funds for responding to Loss & Damage, as well as the requirements for evidence to access these funds, must be viewed through the lens of climate justice. A justice-based approach to Loss & Damage centers on those most at risk, ensures their participation, protects their rights, and strengthens their knowledge systems.

Bridging climate change attribution studies with Indigenous Peoples and Local Communities’ fire knowledge is a promising way forward. Attribution refers to the process of analyzing the relative roles of multiple causal factors to a change or event, along with an assessment of confidence^10^. Attribution studies can quantify the extent to which human-induced climate change influences wildfire likelihood and severity, helping to build the evidence base for fair Loss & Damage allocations. Meanwhile, Indigenous Peoples and Local Communities’ fire practices and associated ecological knowledge offer long-standing, place-based, and contextual strategies for rethinking approaches in a rapidly changing world. Examples of how Indigenous Peoples and Local Communities’ knowledge can be incorporated into attribution studies include integrating in fire modeling traditional burning practices for wildfire risk reduction^3^ and considering traditional on-ground experiences and perceptions of fire drivers and impacts to inform future climate projections, some of which are reported in Box 1. Integrating these two knowledge systems can inform equitable, effective, and locally appropriate responses.

Incorporating dedicated mechanisms and funding for wildfires within Loss & Damage is not only overdue, but also essential. We suggest that without practical, low-barrier mechanisms for funding access, the disproportionate impacts of fire on the most vulnerable communities will persist and continue to deepen existing inequalities in climate governance. Recognition of wildfires as one of the central drivers of climate-related losses and damages, and their inclusion in international frameworks, is a critical step toward climate justice in a warming world.

Box 1 During a meeting in June 2025 in Brasilia (Brazil) with volunteer and community brigades, and during a visit in August 2025 to Caititu Indigenous Land in the Amazonas state, both conducted within the scope of the workshop, community members reported climate change impacts they have been perceiving Over the past decade, they have experienced longer and more intense dry seasons and less intense rainy seasons. In Pantanal, community members reported a shift in fire season to a month earlier, whereas in the Amazon, fire season is shifting to a month later. Accidental or criminal fires lose control more rapidly.

These not only cause immediate impacts but also changes in long-standing cultural practices. For example, traditional fire practices are now conducted at a different time of year. This affects the quality of harvests and, consequently, food access. In addition, smoke not only exacerbates health problems and reduces air quality but also reaches rivers, contaminating fish and further threatening food security.

Visit to Caititu Indigenous Land, in Amazonas state, in August 2025 where the participatory workshop was held. Image credits: Renata M. da Veiga.
